# tRNA abundance, modification and fragmentation in nasopharyngeal swabs as biomarkers for COVID-19 severity

**DOI:** 10.3389/fcell.2022.999351

**Published:** 2022-11-01

**Authors:** Christopher D. Katanski, Hala Alshammary, Christopher P. Watkins, Sihao Huang, Ana Gonzales-Reiche, Emilia Mia Sordillo, Harm van Bakel, Mount Sinai PSP study group, Shelcie Fabre, Karen Lolans, Viviana Simon, Tao Pan

**Affiliations:** ^1^ Department of Biochemistry and Molecular Biology, University of Chicago, Chicago, IL, United States; ^2^ Department of Microbiology, Icahn School of Medicine at Mount Sinai, New York, NY, United States; ^3^ Center for Vaccine Research and Pandemic Preparedness (C-VARPP), Icahn School of Medicine at Mount Sinai, New York, NY, United States; ^4^ Department of Genetics and Genomic Sciences, Icahn School of Medicine at Mount Sinai, New York, NY, United States; ^5^ The Global Health and Emerging Pathogens Institute, Icahn School of Medicine at Mount Sinai, New York, NY, United States; ^6^ Department of Pathology, Molecular and Cell-Based Medicine, Icahn School of Medicine at Mount Sinai, New York, NY, United States; ^7^ Icahn Genomics Institute, Icahn School of Medicine at Mount Sinai, New York, NY, United States; ^8^ Department of Medicine, University of Chicago, Chicago, IL, United States; ^9^ Division of Infectious Diseases, Department of Medicine, Icahn School of Medicine at Mount Sinai, New York, NY, United States

**Keywords:** tRNA, modification, fragmentation, biomarker, SARS-CoV-2, COVID-19 severity

## Abstract

Emerging and re-emerging respiratory viruses can spread rapidly and cause pandemics as demonstrated by the recent severe acute respiratory syndrome coronavirus 2 (SARS-CoV-2) pandemic. The early human immune responses to respiratory viruses are in the nasal cavity and nasopharyngeal regions. Defining biomarkers of disease trajectory at the time of a positive diagnostic test would be an important tool to facilitate decisions such as initiation of antiviral treatment. We hypothesize that nasopharyngeal tRNA profiles could be used to predict Coronavirus Disease 19 (COVID-19) severity. We carried out multiplex small RNA sequencing (MSR-seq) on residual nasopharyngeal swabs to measure simultaneously full-length tRNA abundance, tRNA modifications, and tRNA fragmentation for the human tRNA response to SARS-CoV-2 infection. We identified distinct tRNA signatures associated with mild symptoms versus severe COVID-19 manifestations requiring hospitalization. These results highlight the utility of host tRNA properties as biomarkers for the clinical outcome of SARS-CoV-2.

## Introduction

A major response of biological systems to environmental change is regulated protein synthesis where transfer RNA (tRNA) plays a key role in decoding genetic information on-demand. tRNAs are small non-coding RNAs (74–95 residues) that read the genetic code and provide amino acids in protein synthesis. A human cell has several hundred tRNA sequences, up to 100 million tRNA transcripts, and each human tRNA contains on average 13 modifications (Chan and Lowe, 2016; Boccaletto et al., 2022). Modifications in tRNAs are dynamically regulated during cellular stress (Begley et al., 2007; Gu et al., 2014; Zhang et al., 2022) and can affect decoding speed and accuracy of translation. Hypo-modified tRNAs can also become better substrates for RNase cleavage leading to tRNA fragment generation (Huang and Hopper, 2016; Oberbauer and Schaefer, 2018). tRNA fragments are a family of small RNAs that participate in many regulatory processes at the cellular and organismal levels (Anderson and Ivanov, 2014; Schimmel, 2018; Pandey et al., 2021). In the context of viral infections, tRNAs as well as tRNA fragments may facilitate viral replication (Jin and Musier-Forsyth, 2019; Nunes et al., 2020).

Understanding the determinants of severity of viral infections is important for selecting the appropriate level of clinical care including initiation of therapeutic interventions aimed at preventing severe outcomes. In the case of emerging viral pathogens such as severe acute respiratory syndrome coronavirus-2 (SARS-CoV-2), a major challenge is the wide range of Coronavirus Disease 19 (COVID-19) severities. Many viral and host factors influence the symptom severity upon infection, including age, co-morbidities, host genetics and possibly SARS-CoV-2 variant. However, it remains difficult to predict which COVID-19 patients will develop mild or severe symptoms at the time of diagnosis.

Activated immune cells known as granulocytes secrete RNases as part of the innate immune response. In humans, eight secreted RNase genes have been identified, each with a range of antiviral, antibacterial and/or cytotoxic functions (Koczera et al., 2016; Lu et al., 2018). We propose that tRNAs, due to their high abundance and RNase resistance, represent one of the best opportunities to detect early immune activation in the context of viral infection by measuring their ongoing fragmentation, alteration in abundance and in modification levels. These measurements can be made directly from virally lysed or apoptotic epithelial tissues, which often represent the sites of initial respiratory viral infection.

Here we apply Multiplex Small RNA-seq [MSR-seq, (Watkins et al., 2022)] to identify tRNA-based biomarkers to predict SARS-CoV-2 infection severity at the time of a positive test. MSR-seq simultaneously measures tRNA abundance, fragmentation and modifications in a single sequencing library generated directly from RNA isolated from the viral transport media of the diagnostic nasopharyngeal swab. Our results show that multiple tRNA properties vary significantly among individuals with COVID-19 who develop no/mild versus severe symptoms, thus indicating the biomarker potential of human nasal tRNA responses to an infectious disease such as COVID-19.

## Materials and methods

### Ethics statement

The Mount Sinai Pathogens Surveillance Program (MS-PSP) systematically collected residual nasopharyngeal swab samples that tested positive for respiratory pathogens (e.g., SARS-CoV-2, Influenza A virus) after clinical testing was completed. A subset of representative specimen was selected for viral genome sequencing in the context of precision surveillance (Gonzalez-Reiche et al., 2020; Javaid et al., 2021) and remaining RNA was included in this study. The residual diagnostic specimen used in this study were collected during the first wave of the pandemic (March–May 2020) when ancestral SARS-CoV-2 variants were circulating (Gonzalez-Reiche et al., 2020). The Institutional Review Board of the Icahn School of Medicine at Mount Sinai reviewed and approved MS-PSP (13-00981) as well as this study (21-01934).

### Total RNA extraction from viral transport media

Total RNA was extracted from viral transport media (VTM) of the nasopharyngeal swabs using high-throughput specimen processing (KingFisher Flex Purification System, ThermoFisher, cat. 5400610). The MagMax mirVana Total RNA Isolation Kit (ThermoFisher, cat. A27828) was used to extract total RNA from 250 μL of viral transport medium, as per the manufacturer’s protocol. The total nucleic acid concentration measured by nanodrop absorbance ranged from 4–40 ng/μL.

### RNA sample selection

We selected for this study 56 RNA samples extracted from nasopharyngeal swabs that tested positive for SARS-CoV-2, four RNA samples from nasopharyngeal swabs that tested positive for Influenza A, and five RNA samples from individuals without any known viral infection (uninfected control). All SARS-CoV-2 tested samples were collected during the first wave of the SARS-CoV-2 pandemic in New York City (March–May 2020). Thirty-five RNA samples were extracted from VTM of nasopharyngeal swabs collected from individuals who tested positive for SARS-CoV-2 but did not require hospitalization at the time of testing (Group I) while 21 RNA samples were extracted from VTM of nasopharyngeal swabs collected from individuals who were admitted to the hospital due to severe COVID-19 manifestations at the time of testing (Group II).

### Multiplex small RNA sequencing library construction

MSR-seq library construction followed the same procedure as described previously (Watkins et al., 2022). A total of 10 µL of each extracted sample described above were used for sequencing library construction. The key features of MSR-seq are to first ligate total RNA with a bar-coded capture hairpin oligonucleotide which enables pooling of up to 12 bar-coded samples for all subsequent steps. The specific design of the capture hairpin oligo allows for all subsequent steps to be carried out on magnetic streptavidin beads. The basic steps of library construction include 1) deacylation to remove charged amino acids to tRNA; 2) 3’ end repair to remove all 3’ phosphate/cyclic phosphate; 3) first ligation of the barcoded hairpin oligonucleotide; 4) sample pooling, mix with streptavidin beads, wash; 5) remove 3’ phosphate of the capture hairpin oligo; wash; 6) reverse transcription using a thermophilic RT and overnight extension; 7) remove RNA; 8) periodate oxidation to block unligated hairpin oligonucleotide; 9) second ligation of PCR primer; 10) PCR using Illumina index primers.

For low abundant RNA samples, a gel purification step of the final PCR products was added to remove the excess primer only products as follows. After PCR, amplicons were concentrated using the Zymo DNA Clean & Concentrate spin columns (7 equivalents of DNA binding buffer to 1 equivalent of PCR reaction; e.g. 350 µL of DNA binding buffer to 50 µL PCR reaction). Samples were eluted in 12 µL of deionized, autoclaved water.

After eluting off the column, samples were mixed with 2.5 µL of 6x TriTrack gel loading dye and loaded onto a 6% Novex TBE gel. Samples, together with dsDNA size markers were electrophoresed at 180 V for approximately 40 min. After electrophoresis, the gel was incubated for 10 min in 1x SYBR Gold and then imaged on a blue light box. Samples were cut from approximately 170–300 bp, whereas the primer alone products containing barcodes and indexes were approximately 140–145 bp.

After cutting, gel fragments were crushed using a 1 ml pipette tip and then 500 µL of gel elution buffer (200 mM KCl, 50 mM KOAc, pH 7). Gel fragments were incubated overnight (12 + hours). After overnight incubation, samples were centrifuged for 10 min at room temperature at 10,000 g^−1^. The supernatant was then collected and the samples were then centrifuged again to remove any remaining gel fragments. Next, 1 µL of GlycoBlue and 500 µL of isopropanol were added to each tube and the tubes were then placed in a −80°C freezer for a minimum of 1 h.

After cold incubation, the samples were centrifuged at 4°C for 1 h at 17,000 g^−1^. The supernatant was then removed and the remaining pellet was resuspended in 20 µL of deionized, autoclaved water.

### Data analysis

Read processing and mapping: Libraries were sequenced on Illumina Nova-seq S(1)-200) platform, 100 bp paired-end. First, paired end reads were split by barcode sequence using Je demultiplex with options BPOS = BOTH BM = READ_1 LEN = 4:6 FORCE = true C = false 6. Next read 2 files were used to map with bowtie2 with the following parameters: q -p 10 --local—no-unal. Reads were mapped to curated hg19 list of non-redundant tRNA genes with tRNAScan score >40 (Lowe and Chan, 2016). Bowtie2 output sam files were converted to bam files, then sorted using samtools (Danecek et al., 2021). Next IGV was used to collapse reads into 1 nt window. IGV output. wig files were reformatted using custom python scripts (available on GitHub at https://github.com/ckatanski/MSR-seq). The bowtie2 output Sam files were also used as input for a custom python script using PySam, a python wrapper for SAMTools (https://github.com/pysam-developers/pysam) to sum all reads that mapped to each gene. Data was visualized with custom R scripts (available on GitHub at https://github.com/ckatanski/CovidNasalSwabs_2022ck).

tRNA fragmentation: tRNA fragments were identified as previously described (Watkins et al., 2022). Briefly, the precise 3’ end of the read in the MSR-seq procedure represents the 3’ end of the RNA present in the sample. We binned the ends with 3’ ends mapped to the individual tRNA genes between nucleotides 20–30, 30–40, 40–50, 50–60 and >60 (full-length). These bins roughly correspond to the stem loops in tRNA structure and can be used to characterize the broad types of tRNA fragment. The fraction of a fragment was calculated by comparing the number of reads in one bin compared to all the reads in every bin for a particular gene from a particular patient. To summarize the data, the number of reads that sort to each bin among all genes was summed for each patient.

tRNA abundance: Relative abundance of individual tRNA isodecoders was normalized to the 5.8S rRNA reads within each sample. This normalized abundance was then compared between patient groups.

tRNA modification: Mutation rates from bowtie mapping at individual sites were used to estimate modification rates. Analysis focused on well characterized sites with known modifications. Mutation rate is not a 1-to-1 output for modification fraction, but it is known to vary linearly. Thus relative changes are a reliable metric for relative changes in modification levels. Analysis was limited to sites and samples with >50 reads and a >2% mutation rate. After initial site selection, the 2% mutation filter was relaxed for individual site analysis so as to include samples which may have been excluded from initial screening.

*p*-value calculations: To identify differentially expressed, fragmented, or modified tRNAs, patient group comparisons for individual genes were performed with pairwise two-sided t-tests with no correction. To normalize tRNA abundance reads among patients, all abundances were normalized to the well detected 5.8S rRNA reads within the same sample. This normalized abundance was calculated for every tRNA gene using all sense-mapped reads. Comparing abundance of an individual gene between patient groups was done with a two-sided pairwise *t*-test with no correction. Analysis was restricted to isodecoders and samples with >10 reads for the respective tRNA sequence. To compare tRNA fragmentations, reads were subdivided based on the position of the 3’ end mapping. With MSR-seq, this is a faithful representation of the biological 3’ end of the tRNA and can separate fragments from full length tRNAs. Calculation was restricted to only sense reads, and fragments with >10 reads. Notably, similar analysis cannot be done for the 5’ end since truncated 5’ ends could reflect biological fragments or premature termination of reverse transcription in sequencing library preparation. To compare tRNA modifications among patient groups, we calculated the mutation rate at every base for every gene between patient groups using a two-sided pairwise *t*-test with no correction, at a filter of >50 reads per site.

Logistic regression (LR) models and ROC curves: The samples with non-NULL values in the selected features were used to build the LR models (McKinney, 2010; Pedregosa et al., 2011; Harris et al., 2020). The data were shuffled and normalized. Then, 3-fold cross-validation was performed with abundance (rpm/5.8S rpm) of tRNA^iMet^-c1t32, mutation rate at position 9 for mitochondrial tRNA^Val^, fragmentation calculated with tRNA^Arg^(ACG) isoacceptor family in the 30–40 bin or a combination of the three biomarkers and the scores of each sample are predicted. Specificity, sensitivity and AUC (area under curve) were calculated (Pedregosa et al., 2011) and the ROC curves drawn (Hunter, 2007) with python.

Clustering heatmap: The biomarkers with non-NULL values in at least 50% of the mild symptom samples and 50% of the severe symptom samples were selected. For the 68 selected biomarkers, NULL values were filled by the median of all the samples in the mild or severe groups respectively. Then, the data were normalized. The samples were ordered by their 3-fold cross validation scores in the LR model (McKinney, 2010; Pedregosa et al., 2011; Harris et al., 2020) with 68 selected biomarkers in the heatmap (Michael Waskom et al., 2017). The selected biomarkers were clustered in the heatmap.

## Results and discussion

We analyzed the tRNA profile (abundance, modification and fragmentation) using total RNA extracted from residual diagnostic specimen collected from individuals with symptoms suggestive of upper respiratory tract infection who tested positive for SARS-CoV-2. These samples were collected during the first wave of the pandemic when only ancestral SARS-CoV-2 variants were circulating. These biospecimen are of low-biomass and contain only very small amounts of RNA. Using a new library construction technology for tRNA sequencing that uses total RNA from any biological source and on-bead library construction (Watkins et al., 2022), we obtained tRNA-seq data informing simultaneously on full-length tRNA abundance, certain tRNA modifications, as well as 5’ tRNA fragments (5’tRF). After library construction and sequencing we found that RNAs could be mapped to tRNA and other small RNAs at appreciate rates (Supplementary Table S1) with average mapped read counts of 1,006,000, 131,000, 1,102,500 for all small RNA and 397,500, 77,100, 285,000 for tRNA of the uninfected, influenza, and SARS-CoV-2 infected samples, respectively. While these rates are lower than tRNA-seq from cell cultures, they still reveal the potential to obtain high quality small RNA sequencing data from viral transport media of nasopharyngeal swabs used for diagnostic nucleic acid amplification testing (NAAT). Ultimately RNA-seq would be too costly and time-consuming to serve as a practical tool for triage, however our goal was to identify potential prognostic biomarkers for further development using scalable nucleic acid technologies such as qPCR.

**FIGURE 1:**
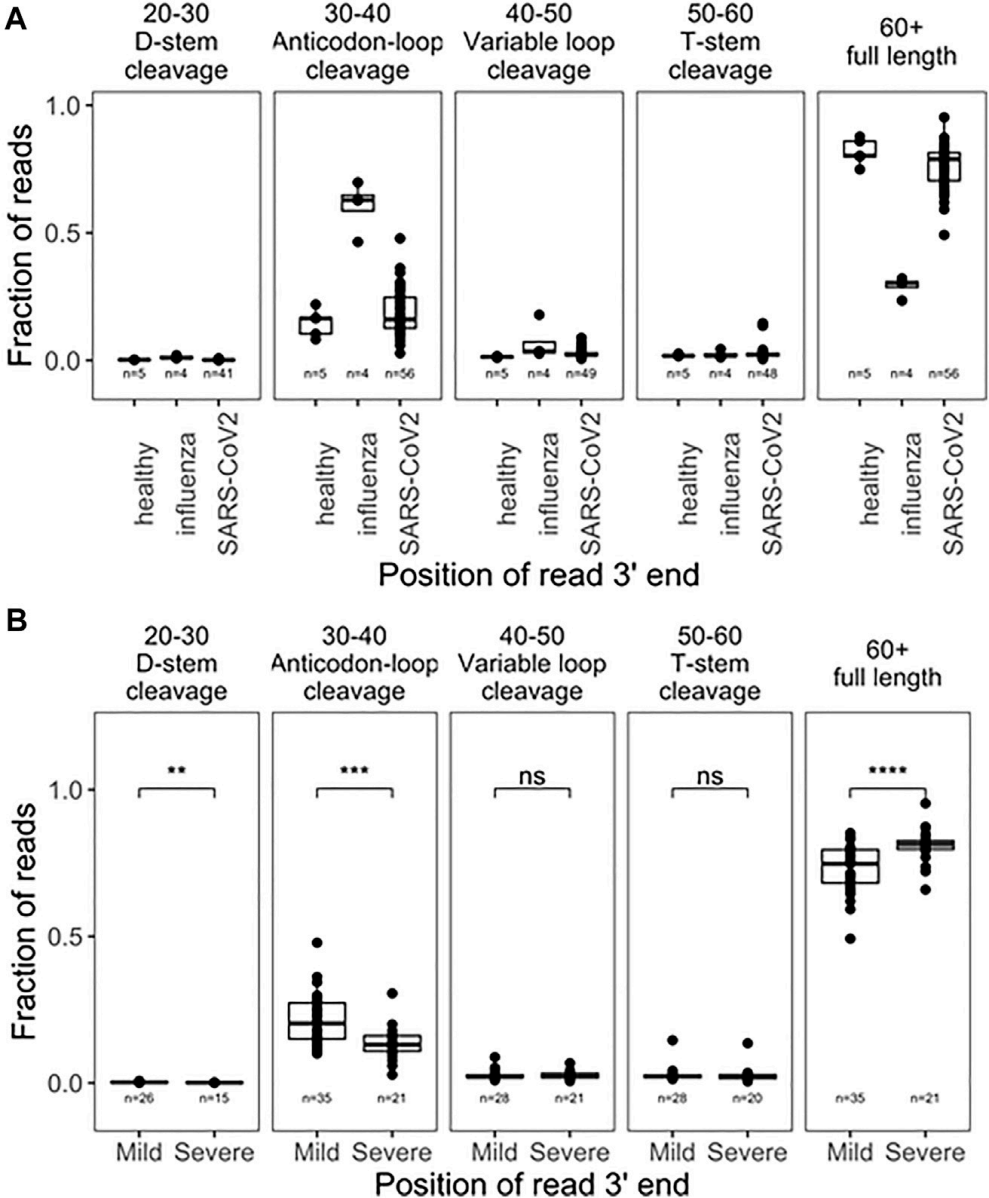
Global tRNA fragmentation pattern among nasopharyngeal swab samples. **(A)** Fraction of reads mapped to different tRNA fragments are shown for uninfected control (*n* = 5), influenza infected (*n* = 4), and SARS-CoV-2 in fected (*n* = 56) individuals. All tRNA species are pooied together to reflect global levels of tRNA fragmentation. **(B)** Comparison of global tRNA fragmentation between SARS-CoV-2 infected patients who go on to develope either mild or severe COVID19 symptoms. All tests are two-side-test with the number of samples passing filters indicated on that plot,*p*-values:*<0.05; **<10^−2^;***<10^−3^;****<10^−4.^

We first analyzed 5’tRF differences among the SARS-CoV-2, influenza, and uninfected groups by binning the tRNA reads with the 3’ end in different tRNA positions [(Watkins et al., 2022), Figure 1A]. Approximately 85% of the tRNA reads had 3’ ends past nucleotide 60 which roughly corresponded to the full-length tRNAs in our analysis. tRNA fragmentation occurred extensively in all samples, compared to well-controlled samples from cell culture (Watkins et al., 2022). Globally, fragmentations of tRNAs were present with cleavage sites in all tRNA regions. As expected, cleavage in the anticodon loop region (3’ ends within position 30–40 of tRNA, anticodon positions approximately 34–36) generated the highest amount of tRF products. Since fragmentation was not evenly distributed among accessible loop regions, this suggests some level of biological specificity. Influenza-infected patients showed a much greater degree of fragmentation compared to healthy subjects, though only 4 samples were available for analysis, limiting the strength of this observation. SARS-CoV-2 infected patients also showed greater fragmentation compared to healthy subjects. Interestingly, SARS-CoV-2 patients who developed mild symptoms showed a significantly higher degree of global tRNA fragmentation compared to patients who developed severe symptoms (Figure 1B). We speculate that this could reflect the strength of an innate immune response to viral infection, which is known to include secretion of human genome-encoded RNases. In such a narrative, patients with weaker immune responses might secrete less defensive RNases, experience less tRNA fragmentation, and develop more severe symptoms.

**FIGURE 2:**
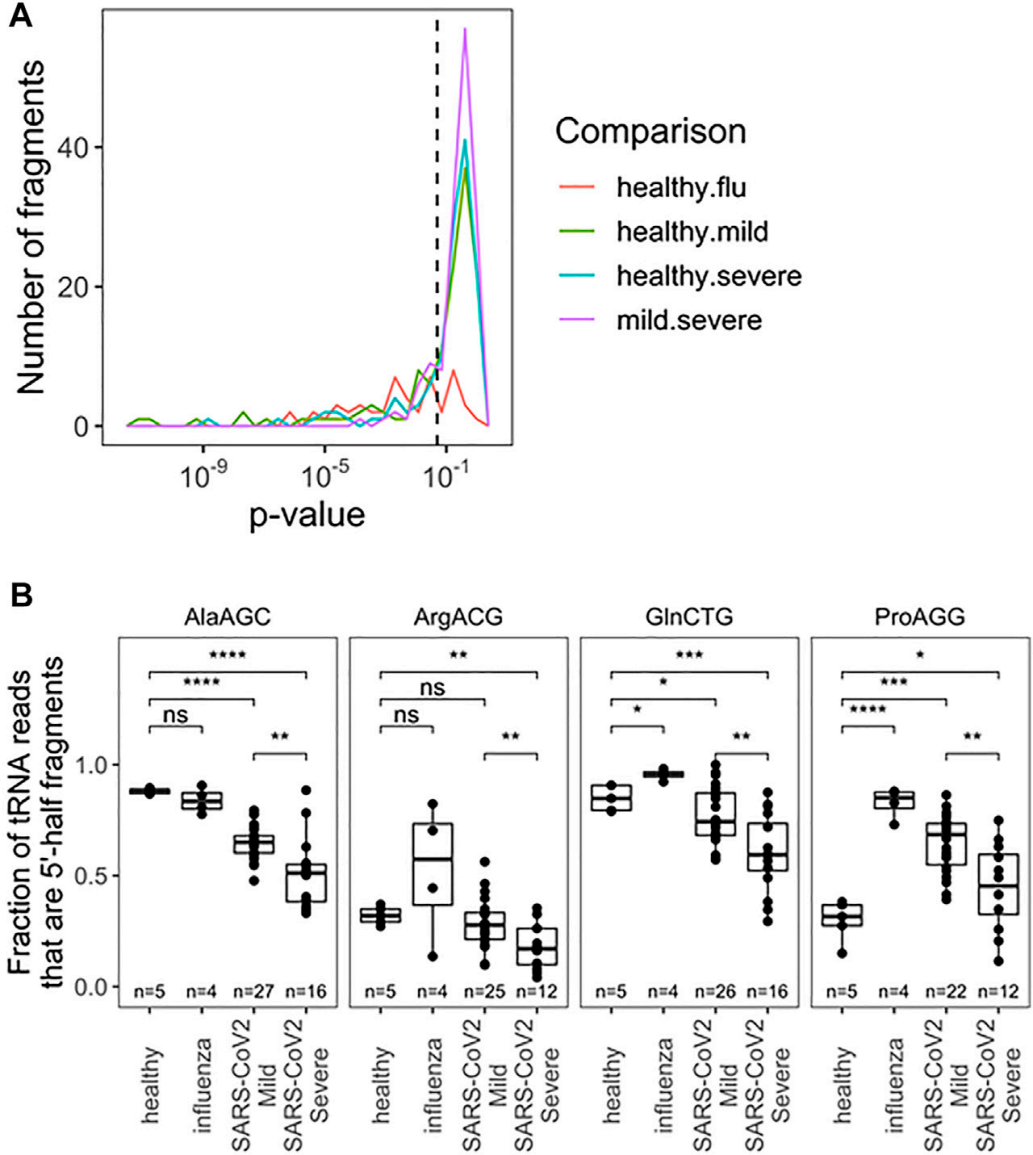
Fragmentation profile of specific tRNA Isoacceptor families allows for seperation of RNA obtained from nasophary swabs collected from SARS-CoV-2 infected individuals who developed mild or severe symptoms. **(A)** Uncorrected *p*-Values for pairwise two-sided t-tests indicate several notable differences in fragmentation amoung specific tRNA isoacceptor families. Precise values are indicated in table S2. **(B)** Fragmentation profies for top anticdon-cleaved tRNAs are hightlighted:tRNA^Ala^, tRNA^Arg^(ACG), tRNA^pro^(AGG), and tRNA^GIn^(CTG). In addition to distinguising among SARS-CoV-2 patients, fragmentation can provide diagnostic insight which compared to healthy patients. *p*-values:*<0.05; **<10^−2^;***<10^−3^;****<10^−4^. Two-side t-tests with the number of samples passing a 10 read fliter indicated in each plot.

We further analyzed the tRF products of specific tRNAs among all pairwise comparisons of patient groups; samples were filtered for fragments with >10 reads (Figure 2A; Supplementary Table S2). Due to limited cohort sizes, we restricted further analysis to the SARS-CoV-2 positive biospecimen. For this analysis, reads from individual isodecoders were pooled among isoacceptor families. This was done because fragment reads often cannot be distinguished among related isodecoder sequences, since the distinguishing bases were cleaved away. Further, RT-qPCR-base assays that can distinguish closely related isodecoders represent an additional challenge, so pooled analysis may yield more transferable insights. Among tRFs of isoacceptor families, we found statistically significant differences among 20 distinct fragments (*p* < 0.05), including fragments for the anticodon loop, variable loop, and T-stem loop (Supplementary Table S2). Fragments from the anticodon loop tend to be more abundant and thus better candidates for development into prognostic qPCR tests. Top candidates include tRNA^Ala^(AGC), tRNA^Arg^(ACG), tRNA^Pro^(AGG), and tRNA^Gln^(CTG) (Figure 2B). For these tRNA isoacceptors, patients who developed mild COVID-19 symptoms produced more tRFs than patients who developed severe COVID-19 symptoms. Again, these results may potentially be associated with the differential activities of immune response in the nasopharyngeal region, for example, the amount of RNase released upon SARS-CoV-2 infection.

**FIGURE 3:**
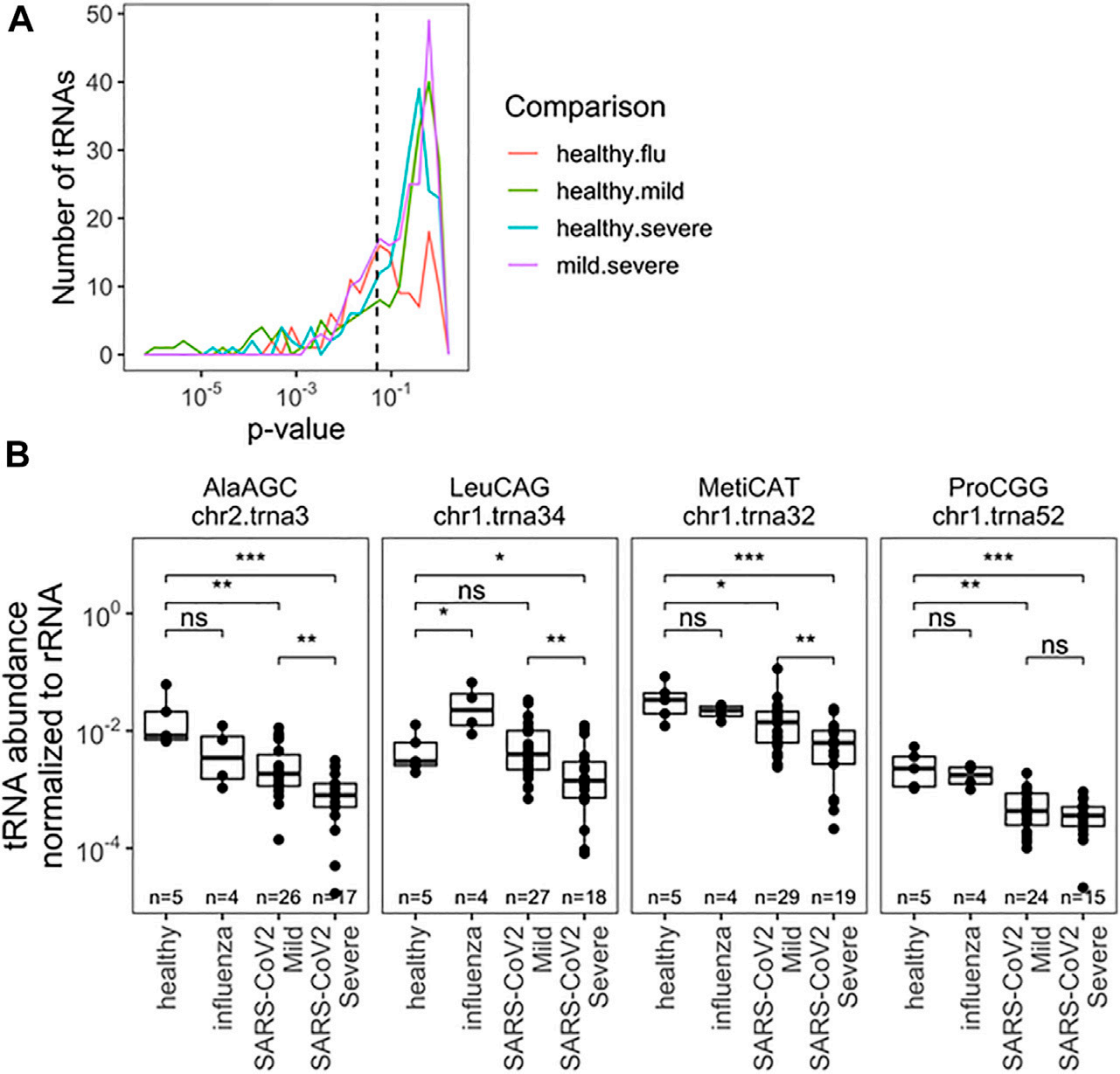
Relative abundance of specific tRNA isodecoders to 5.8S rRNA in the same RNA samples allowes for separation by infection status (uninfected, influenza and SARS-CoV-2 infected individuals). **(A)** Uncorreced *p*-Values for pairwise two-sided t-tests indicate several notable differences in rRNA-normalized abundance among specific. tRNA isodecoders. Analysis inciudes all sense-mapped reads for each isodecoders. Precisee values are indicated in Supplementary Table S3. **(B)** rRNA-normalized abundance for notable abundance tRNAs are highlighted tRNA^Ala^(ACG),c2t3tRNA^Mct^(CAT), and tRNA^Leu^(CGG)c1t34. In addition to distinguisting among SARS-Cov-2 patients, normalized abundance of tRNA^Pro^(CGG)c1t52. amoung others, can provide diagnostic insight which compared to healthy patients.*<0.05; **<10^−2^;***<10^−3^;****<10^−4^. Two-side *t*-tests with the number of samples passing a 10 read fliter indicated in each plot.

Next, we analyzed the abundance of full-length tRNAs at the isodecoder level. Abundance was measured relative to 5.8S rRNA, a very abundant small rRNA present in all samples; isodecoders were limited to samples with >10 reads. This choice was made with development of scalable prognostics in mind, where normalization must be done verses a specific, measurable RNA species, as opposed to a “reads per million” approach for typical of RNA-seq analysis. The abundance of all tRNA reads, summed together, compared with 5.8S rRNA was different between uninfected, influenza and SARS-CoV-2 infected groups, though not between SARS-CoV-2 patients (Supplementary Figure S1). This may reflect global changes in translation activity within cells or differences in the nature of collected material (e.g., lytic cell debris vs. shed cells). This result also highlights the critical importance of choosing normalization standards. We found significantly different levels of specific tRNA isodecoders in all pairwise comparisons, but restricted our analysis to the two SARS-CoV-2 groups with the most biospecimen and no global differences in total tRNA abundance. Here 53 isodecoders showed significant abundance differences between the mild and severe SARS-CoV-2 groups (Figure 3A; Supplementary Table S3). We highlight three tRNAs that can distinguish between mild and severe SARS-CoV-2 patient groups (Figure 3B): tRNA^Ala^(AGC)c2t3, tRNA^Met^(CAT)c1t32, and tRNA^Leu^(CGG)c1t34. Further, we highlight tRNA^Pro^(CGG)c1t52 which can be used to distinguish SARS-CoV-2 patients from healthy controls. Interestingly tRNA^Leu^(CGG)c1t34 is not well suited as a general SARS-CoV-2 marker, despite discriminating between symptom groups, demonstrating a diversity of behaviors. Of note, tRNA^Met^(CAT)c1t32 is the main initiator tRNA isodecoder in human cells (Chan and Lowe, 2016) and the observed differences in its abundance may reflect the translation activity of the human nasal cells upon SARS-CoV-2 infection.

**FIGURE 4:**
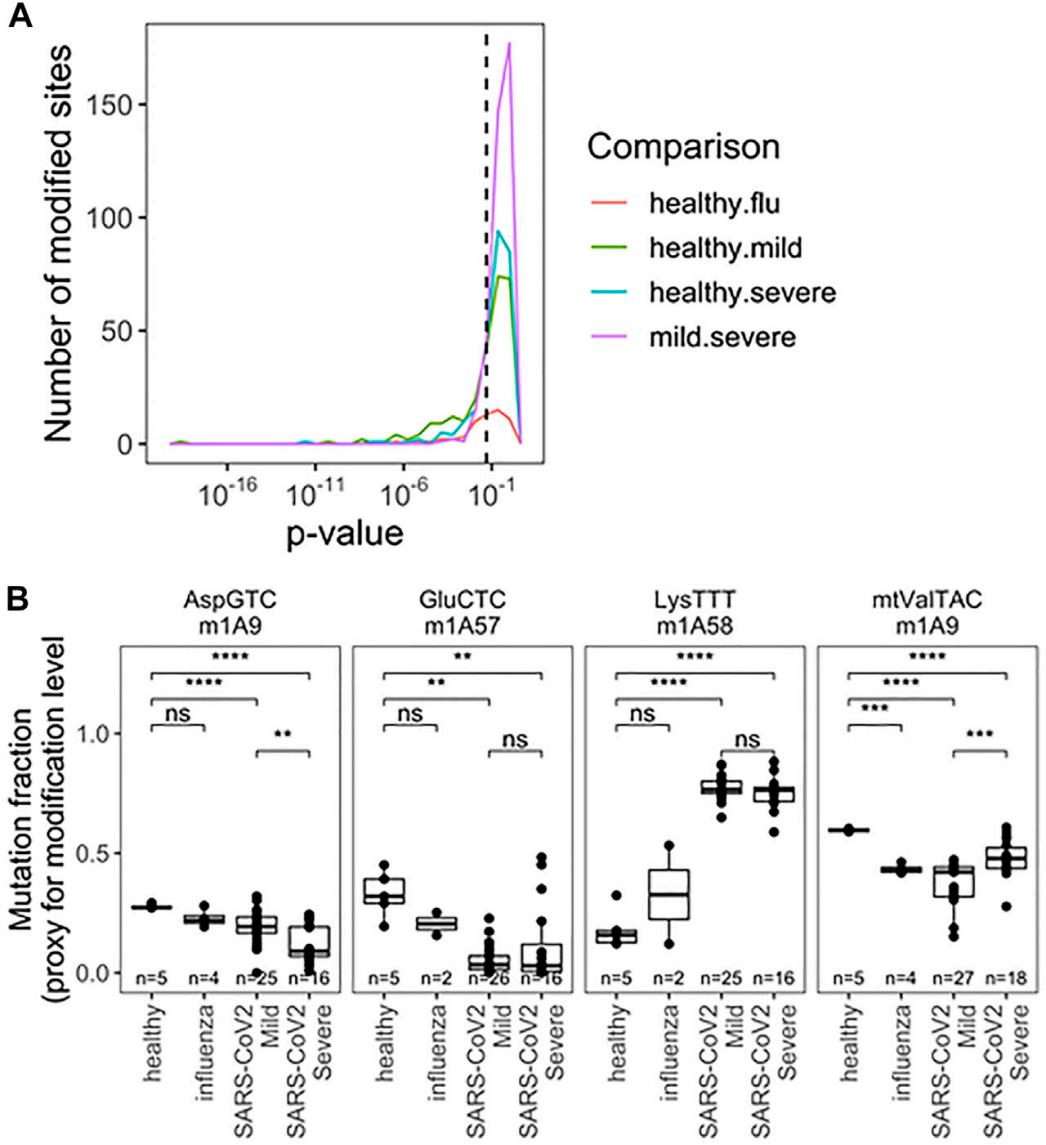
Rspecific tRNA modification profile in RNA obtained fromnasopharyngeal swabs allows for seperation of uninfected, influenza and SARS-CoV-2 infected individuals with mild or severe Covid -19 symptoms). **(A)** Uncorreced *p*-Values for pairwise two-sided *t*-tests indicate differences amoung specific tRNA modifications. Modifications are detected as a “muation” derived from reverse transcriptase disincorporation when reading modified bases. Analysis includes sites with >2% mutation rate and >50 mapped reads. Precise values are indicated in table S4. **(B)** Modifications exhibiting notable patterns are highlighted m^1^A9 on tRNA^Asp^(GTC), m1A58 on tRNA^Glu^(CTC), m1A58 on tRNA^1ys^(TTT),m^1^A9 on mt-tRNA^Val^(TAC). *p*-values.*<0.05; **<10^−2^;***<10^−3^;****<10^−4^. Two-side *t*-tests with the number of samples passing a 50 read fliter indicated on each plot-the 2% mutation rate filter was relaxed after individual sites were chosen.

The final parameter we obtained from our sequencing data was the comparison of tRNA modification levels through RT mutation signatures (Helm and Motorin, 2017). Briefly, certain modifications interfere with reverse transcription during library preparation, leading to enzymatic misincorporation of bases, which is measured as a “mutation” during sequencing. These signatures do not represent DNA-level mutations, but misincorporation by reverse transcriptase. The mutation rate at specific site can be quantitatively compared to access the differences between modification changes between any two samples. This analysis is particularly sensitive to tRNA modifications with the added chemical group at the Watson-Crick face of the nucleobase, for example, N1-methyladenosine [m^1^A, (Cozen et al., 2015; Clark et al., 2016)] which is among the most widespread human tRNA modification types. Using the differences in mutation fractions, we found specific tRNA modifications that can distinguish all pairwise patient groups (Figure 4A). Analysis was limited to sites with a mutation rate >2% and more than 50 reads to limit spurious differences arising from noise at sites known to be unmodified. Again we restrict our analysis to SARS-CoV-2 patients with a greater number of patients sampled. Together, 33 different modification sites showed significant ability to distinguish patient groups, with examples in the D-loop, anticodon loop, and T-stem loop (Figure 4A; Supplementary Table S4). *A priori*, it is not obvious what effect changes in modification level can have on changes in tRNA biology, including abundance and fragmentation. For example, we previously reported that different modifications can either stimulate or protect from tRNA fragmentation (Watkins et al., 2022). Here we observed a variety of behaviors from different modifications relating to SARS-CoV-2 infection (Figure 4B). First, m^1^A9 on tRNA^Asp^(GTC) showed progressive reduction of methylation from healthy, to mild symptoms, to severe symptoms patients. Similarly, methylation of m^1^A58 on tRNA^Glu^(CTC) was reduced drastically in all SARS-CoV-2 positive biospecimen compared to those from healthy controls. This site is not suitable to distinguish between SARS-CoV-2 severity groups, likely because the methylation levels are already fully reduced. The opposite behavior was observed for tRNA^Lys^(TTT) where methylation at m^1^A58 dramatically increased upon SARS-CoV-2 infection, but again, methylation cannot distinguish among SARS-CoV-2 patients. Finally, methylation of m^1^A9 of mt-tRNA^Val^(TAC) followed a pattern remittent of fragmentation: methylation was significantly reduced in samples obtained from healthy controls compared to samples from patients with mild COVID-19. This response was further muted in biospecimen from patients with severe COVID-19. Of note, mitochondrial tRNA^Val^ plays a dual role in mitochondrial translation. It not only works as a tRNA in translation but is also an essential component of the human mitochondrial ribosome (Amunts et al., 2015). M^1^A9 in mt-tRNA^Val^ may, thus, modulate its activity either as a tRNA and/or its ribosomal function. This could plausibly be related to global changes in translation reflected in Supplementary Figure S1.

**FIGURE 5:**
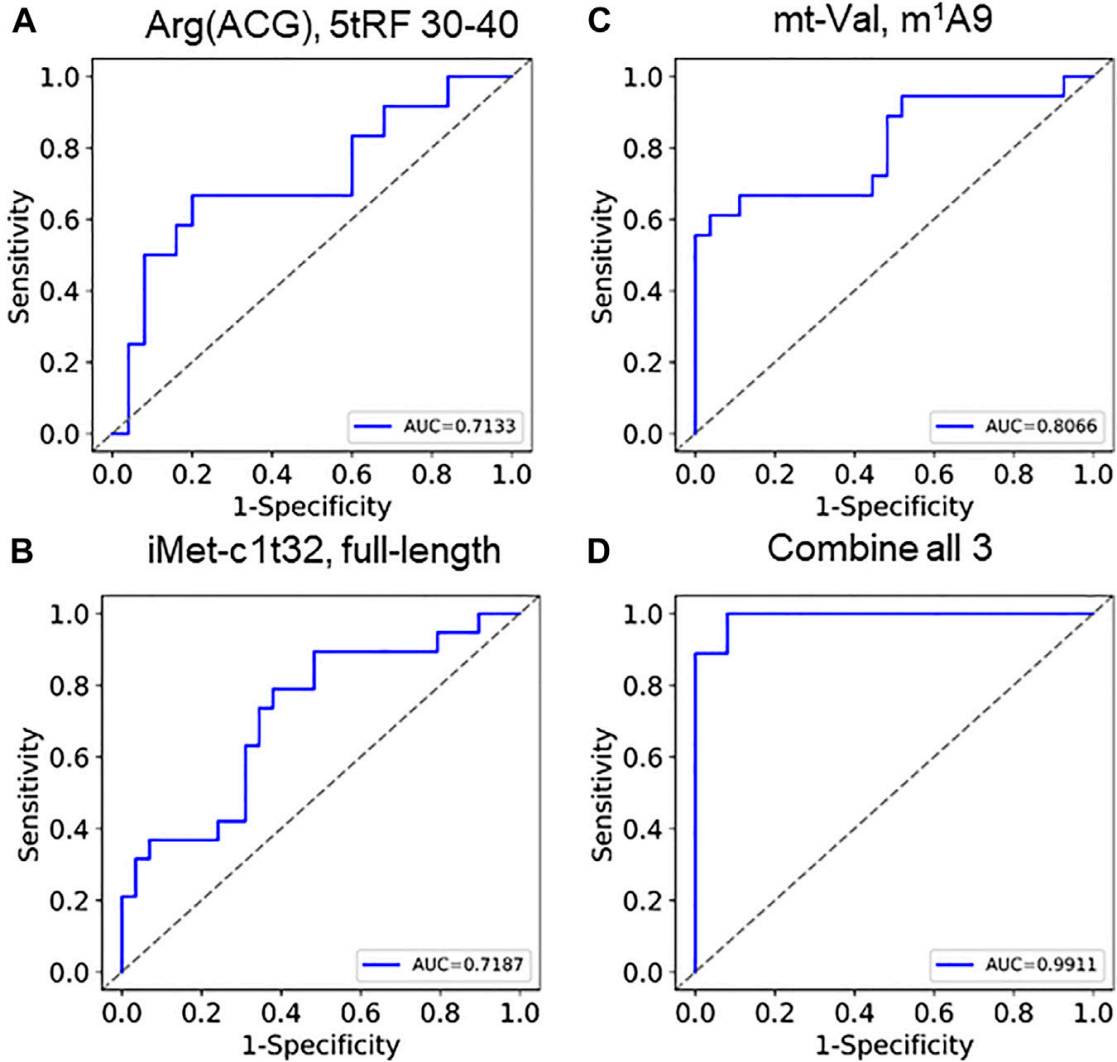
ROC curves of using tRNA abundance, modification, and fragmentation as biomarkers Individual curves of **(A)** fragmentation (tRNA^Arg^(ACG),5′tRF), **(B)** abundance (tRNA_i_^Met^(CAT)c1t32), **(C)** modification (tmitochondrial tRNA^Val^,m^1^A9) show AUC between 0.71 and 0.81. **(D)** Combining these 3 generates an AUC of 0.99.

Based on the findings described above, we perceive that these tRNA features could be used as biomarkers. Clustering 68 selected tRNA biomarkers from 60 samples show that the two SARS-CoV-2 groups are markedly separated (Supplementary Figure S2). As a consideration for future qPCR type biomarker assays, we selected one specific tRNA for fragmentation, abundance, and modification for statistical calculation of COVID-19 severity (Figures 5A–C; Supplementary Figures S3A–C). Each of the three individual parameters produced an area under curve (AUC) value of 0.71–0.81. We developed a combine metric using a linear combination of individual measurements of tRNA abundance, modification, and fragmentation. Using these three tRNA properties, we obtained an AUC value of 0.99 (Figures 5D, Supplementary Figure S3D), indicating the power of using multiple tRNA properties for accurate prediction of SACS-CoV-2 infection symposium severity.

In summary, we were able to generate good quality tRNA-seq results from residual diagnostic nasopharyngeal biospecimen. tRNA profiles of these RNA samples, taken at the time of initial SARS-CoV-2 diagnosis, may provide new information that can be used to predict COVID-19 symptom severity. These results represent a distinct approach in defining biomarkers of infectious disease severity which may allow for the identification of patients at high risk for complication from respiratory virus infection. Future work will test our hypothesis that these tRNA signatures are related to the nasal innate immune RNase secretions and represent non-genetic factors contributing to viral pathogenesis. Future studies are also needed to independently validate these findings and to develop assays to allow for rapid testing in the setting of clinical applications, free from the cost and time constrains of the sequencing-based approach used here. Notably, developing methods scalable that can distinguish tRNA fragments from intact tRNAs are sorely lacking. The best current approaches require gel-based size selection which is labor and bio-mass intensive, and thus not scalable. Similarly, qPCR methods to measure tRNA modifications with a useful dynamic range will be crucial for this nuanced biology to become clinically impactful. Current tRNA-focused RT-qPCR approaches measure an uninterpretable amalgam of full length tRNA, RT-induced stops, and tRNA fragmentation. Here, we articulate the value of developing more precise methods for clinical deployment.

## Mount Sinai Pathogen Surveillance Program (PSP) study group

Shelcie Fabre, Jose Polanco, Matthew M. Hernandez, Alberto Paniz-Mondolfi.

## Data Availability

The datasets presented in this study can be found in online repositories. The names of the repository/repositories and accession number(s) can be found below: All sequencing data have been deposited to NCBI GEO under the accession GSE208612.
