# Supplementary material for: tRNA abundance, modification and fragmentation in nasopharyngeal swabs as biomarkers for COVID-19 severity
Source: Front Cell Dev Biol. 2022 Nov 1;10:999351. doi: 10.3389/fcell.2022.999351 (PMC9664364; doi:10.3389/fcell.2022.999351)
Supplement: Supplementary file 3 [file Presentation1.PPTX]

## Slide 1
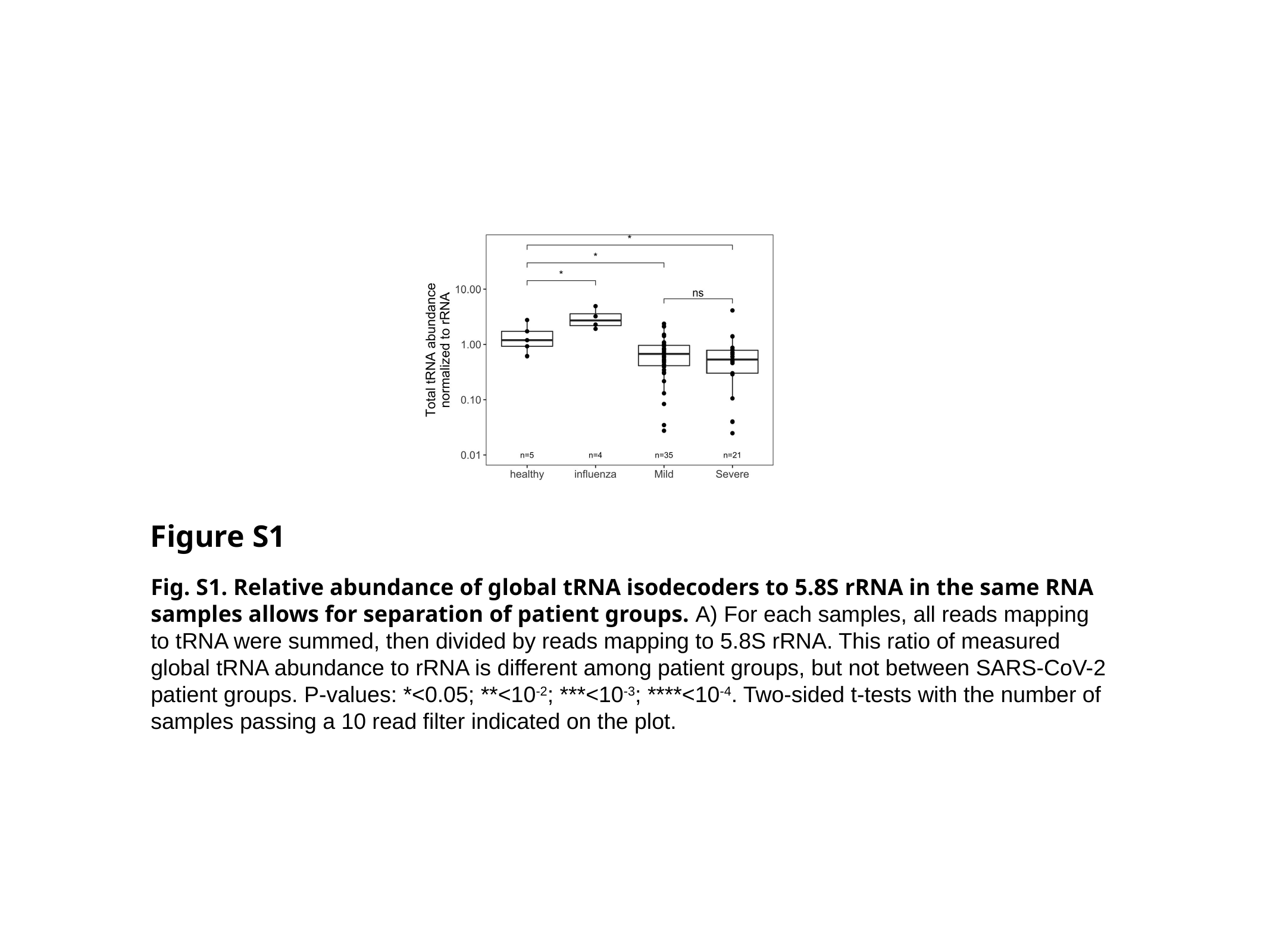

Figure S1
Fig. S1. Relative abundance of global tRNA isodecoders to 5.8S rRNA in the same RNA samples allows for separation of patient groups. A) For each samples, all reads mapping to tRNA were summed, then divided by reads mapping to 5.8S rRNA. This ratio of measured global tRNA abundance to rRNA is different among patient groups, but not between SARS-CoV-2 patient groups. P-values: *<0.05; **<10-2; ***<10-3; ****<10-4. Two-sided t-tests with the number of samples passing a 10 read filter indicated on the plot.

## Slide 2
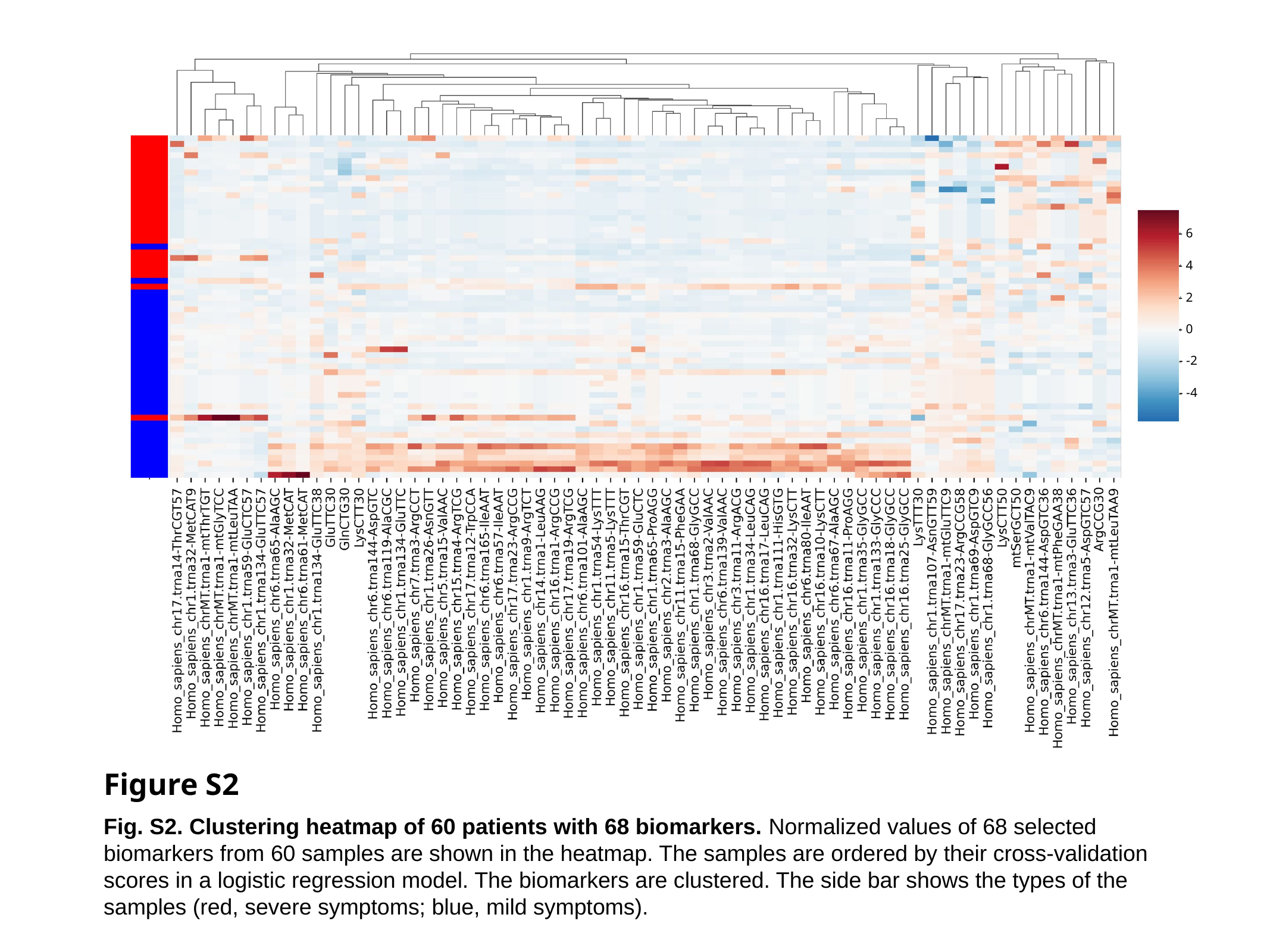

- 6
- 4
- 2
- 0
- -2
- -4
Figure S2
Fig. S2. Clustering heatmap of 60 patients with 68 biomarkers. Normalized values of 68 selected biomarkers from 60 samples are shown in the heatmap. The samples are ordered by their cross-validation scores in a logistic regression model. The biomarkers are clustered. The side bar shows the types of the samples (red, severe symptoms; blue, mild symptoms).

## Slide 3
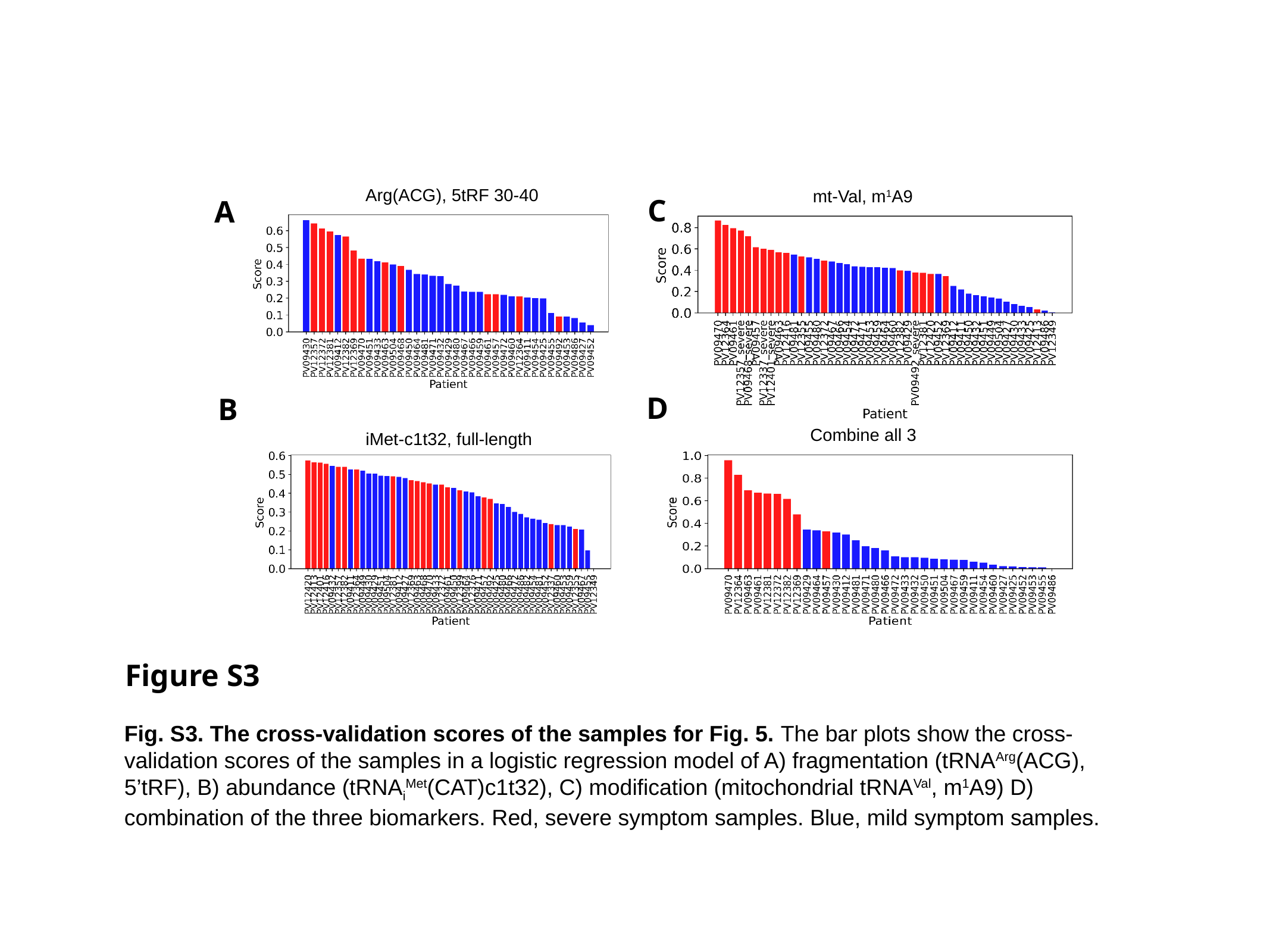

Arg(ACG), 5tRF 30-40
mt-Val, m1A9
C
A
D
B
Combine all 3
iMet-c1t32, full-length
Figure S3
Fig. S3. The cross-validation scores of the samples for Fig. 5. The bar plots show the cross-validation scores of the samples in a logistic regression model of A) fragmentation (tRNAArg(ACG), 5’tRF), B) abundance (tRNAiMet(CAT)c1t32), C) modification (mitochondrial tRNAVal, m1A9) D) combination of the three biomarkers. Red, severe symptom samples. Blue, mild symptom samples.

## Slide 4
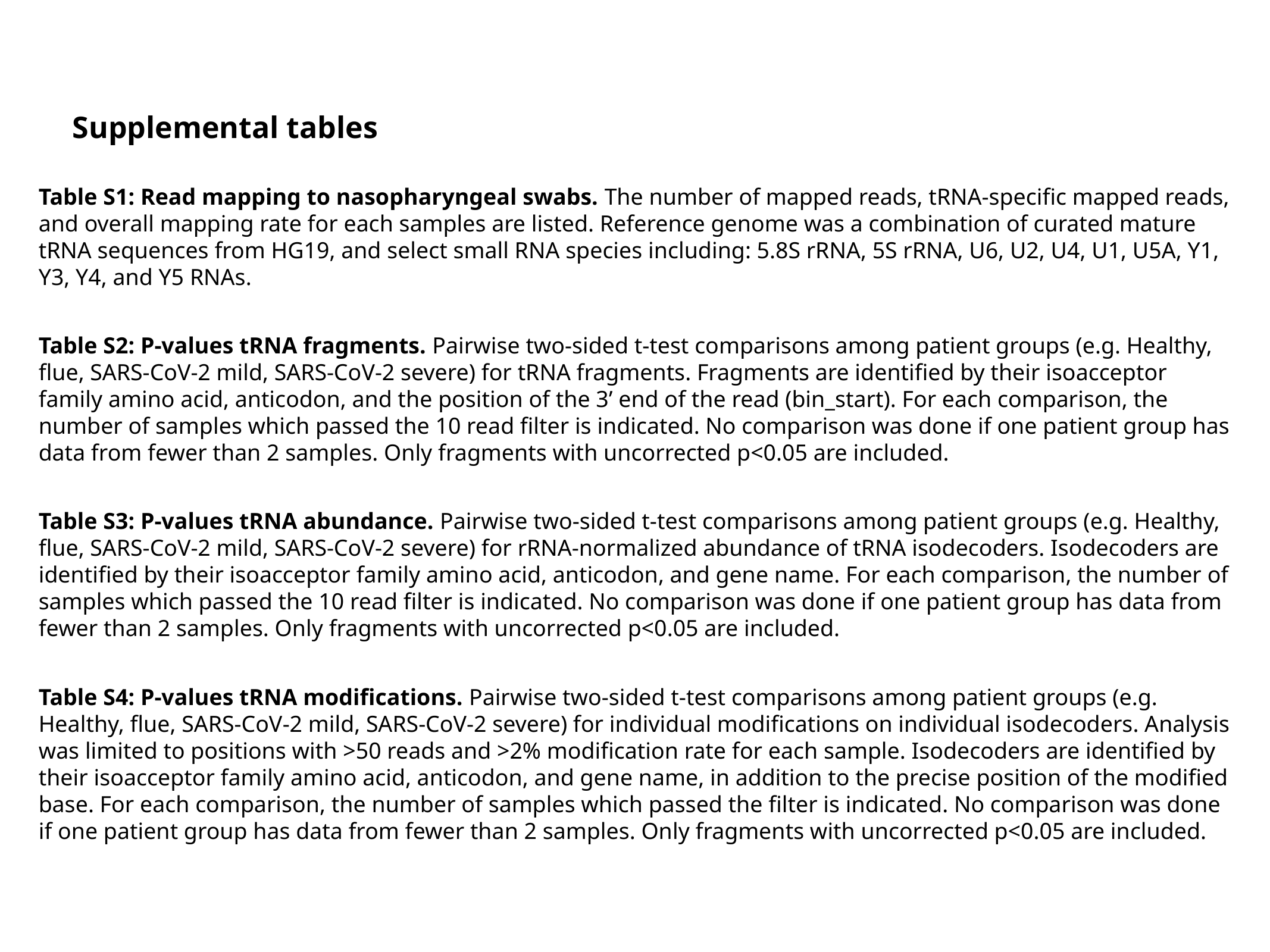

Supplemental tables
Table S1: Read mapping to nasopharyngeal swabs. The number of mapped reads, tRNA-specific mapped reads, and overall mapping rate for each samples are listed. Reference genome was a combination of curated mature tRNA sequences from HG19, and select small RNA species including: 5.8S rRNA, 5S rRNA, U6, U2, U4, U1, U5A, Y1, Y3, Y4, and Y5 RNAs.
Table S2: P-values tRNA fragments. Pairwise two-sided t-test comparisons among patient groups (e.g. Healthy, flue, SARS-CoV-2 mild, SARS-CoV-2 severe) for tRNA fragments. Fragments are identified by their isoacceptor family amino acid, anticodon, and the position of the 3’ end of the read (bin_start). For each comparison, the number of samples which passed the 10 read filter is indicated. No comparison was done if one patient group has data from fewer than 2 samples. Only fragments with uncorrected p<0.05 are included.
Table S3: P-values tRNA abundance. Pairwise two-sided t-test comparisons among patient groups (e.g. Healthy, flue, SARS-CoV-2 mild, SARS-CoV-2 severe) for rRNA-normalized abundance of tRNA isodecoders. Isodecoders are identified by their isoacceptor family amino acid, anticodon, and gene name. For each comparison, the number of samples which passed the 10 read filter is indicated. No comparison was done if one patient group has data from fewer than 2 samples. Only fragments with uncorrected p<0.05 are included.
Table S4: P-values tRNA modifications. Pairwise two-sided t-test comparisons among patient groups (e.g. Healthy, flue, SARS-CoV-2 mild, SARS-CoV-2 severe) for individual modifications on individual isodecoders. Analysis was limited to positions with >50 reads and >2% modification rate for each sample. Isodecoders are identified by their isoacceptor family amino acid, anticodon, and gene name, in addition to the precise position of the modified base. For each comparison, the number of samples which passed the filter is indicated. No comparison was done if one patient group has data from fewer than 2 samples. Only fragments with uncorrected p<0.05 are included.
